# Antimicrobial Peptides—or How Our Ancestors Learned to Control the Microbiome

**DOI:** 10.1128/mBio.01847-21

**Published:** 2021-09-28

**Authors:** Thomas C. G. Bosch, Michael Zasloff

**Affiliations:** a Zoological Institute, University of Kiel, Kiel, Germany; b Georgetown University Hospital, Washington, DC, USA

**Keywords:** microbial communities, microbiota, symbionts, pathogens, plant immune system, microbial ecology, innate immunity, *Hydra*, *Euprymna*

## Abstract

Antimicrobial peptides (AMPs) are short and generally positively charged peptides found in a wide variety of life forms from microorganisms to humans. Their wide range of activity against pathogens, including Gram-positive and -negative bacteria, yeasts, fungi, and enveloped viruses makes them a fundamental component of innate immunity. Marra et al. (A. Marra, M. A. Hanson, S. Kondo, B. Erkosar, B. Lemaitre, mBio 12:e0082421, 2021, https://doi.org/10.1128/mBio.00824-21) use the analytical potential of *Drosophila* to show that AMPs and lysozymes play a direct role in controlling the composition and abundance of the beneficial gut microbiome. By comparing mutant and wild-type flies, they demonstrated that the specific loss of AMPs and lysozyme production results in changes in microbiome abundance and composition. Furthermore, they established that AMPs and lysozyme are particularly essential in aging flies. Studies of early emerging metazoans, other invertebrates, and humans support the view of an ancestral function of AMPs in controlling microbial colonization.

## COMMENTARY

Antimicrobial peptides (AMPs) are known as short, positively charged, and amphipathic peptides with a broad spectrum of antibacterial, antiviral, and antifungal activities. They were initially discovered in mammalian white blood cells (1), insect hemolymph (2), and frog skin (3) and now are considered an inherent part of the animal (and plant) defense system against microbial pathogens. AMPs are present in all classes of life. They are highly diverse within and across species, with most plant and animal genomes encoding 5 to 10 distinct AMP families (4). Given their broad spectrum of activity, much effort has been made to use AMPs as novel antibacterial drug candidates in the campaign against bacterial infection (4). Some of the AMPs such as defensins are evolutionarily conserved across organisms from flies to humans. Other AMPs are restricted to particular clades and therefore considered taxonomically restricted genes (TRGs) (5). Examples include hydramacin, periculin, and arminin in *Hydra* (6) and drosocin, diptericin, and drosomycin in *Drosophila* (7).

## AMPs DIRECTLY REGULATE MICROBIOTA DIVERSITY AND ABUNDANCE IN THE *DROSOPHILA* GUT

Previous studies had shown that in animals in which the Relish transcription factor, the major regulator of the immune deficiency (IMD) pathway, which includes hundreds of genes involved in systemic and local immunity, was deleted, animals died prematurely with increased numbers of gut bacteria, and the species composition of the gut bacteria was different. In this report, Marra et al. (8) focus on the role of the two major antimicrobial effectors expressed in the gut, AMPs and lysozyme, in the management of gut microbiota. Marra et al. (8) created germfree flies by sterilization at the embryonic stage. Two days after reaching adulthood, the flies were provided food containing specific microbial inocula. In one experiment, the animals received a cocktail of six bacterial isolates common to the normal *Drosophila* microbiome. Microbial colonization was analyzed after vertical transmission in the next generation of flies. In a second experiment, flies were fed the same cocktail of microbes, and the gut microbiota was analyzed 10 and 29 days later. In a third experiment, the 2-day-old germfree adult flies were fed monocultures, and gut contents were analyzed 6 days later. The experiments were conducted with wild-type flies, mutants in which the major AMPs had been deleted, mutants lacking the major lysozyme genes, and an IMD mutant.

Marra et al. (8) demonstrated that the AMP mutants resembled the Relish mutants with respect to microbial load and composition, suggesting that much of the disturbance of the gut microbiome observed in IMD mutants can be ascribed to impaired expression of AMPs. Furthermore, flies lacking AMPs or lysozymes displayed reduced microbial community stability and high degree of variability of microbial species. Loss of these effector molecules led to increased microbiota abundance and changes in composition as the flies aged. Thus, the work by Marra et al. (8) demonstrates that both AMPs and lysozymes play a role in establishing and maintaining a stable gut microbiome in *Drosophila*. Marra and colleagues’ innovation is to show that AMPs not only defend the fly from systemic infection but also, with the assistance of lysozyme, help shape the microbiome of its gut.

## CAN ONE GENERALIZE THESE OBSERVATIONS? WHAT ABOUT OTHER SYSTEMS?

Antimicrobial peptides were also shown in a number of other invertebrate species to play an important role in establishing and maintaining a stable microbiome. When studying the natural association between Vibrio fischeri and the Hawaiian bobtail squid, McFall-Ngai and Ruby demonstrated that antimicrobials are used to select V. fischeri to colonize the light organ (9, 10). Upregulation of genes encoding antimicrobials like hemocyanin (11) seems to create a biochemical environment in the mucus that selects for V. fischeri and prepares the cells for the elevated antimicrobial environment of host tissues. Many of the antimicrobials shed with the mucus at hatching, including a peptidoglycan recognition protein and lysozyme, are specific for Gram-positive bacteria, which is likely to contribute to their absence in the aggregates (9).

In the early emerging cnidarian *Hydra*, microbial colonization in early embryos is controlled by maternally encoded antimicrobial peptides (12). After mid-blastula transition, zygotically expressed antimicrobial peptides take control of the microbiome. After hatching, a stable microbiome is established within 3 to 4 weeks (13) which replaces the maternally produced AMPs. In adult *Hydra*, additional AMPs (14, 15) contribute to the host-derived control of bacterial colonization. In addition to epithelial cells, AMPs are also produced by neurons (16). Transgenic polyps which do not express neuron-derived antimicrobial peptide 1 (NDA-1) have a disturbed microbial colonization pattern. NDA-1 contributes to the reduction of Gram-positive bacteria during early development and thus to a spatial distribution of the main colonizer, the Gram-negative *Curvibacter* sp., along the bod*y* axis. Thus, *Hydra* AMPs shape the microbiome and contribute to the spatial organization of the microbiota.

AMPs also are fundamental components of human innate immunity. As in *Drosophila*, the AMPs are components of an armamentarium that includes antimicrobial proteins such as lysozyme, as well as RNases, iron sequestering proteins, and antibacterial lectins. In the eye, skin, airway, and gastrointestinal, reproductive, and urinary tracts, AMPs are secreted onto the surface of the epithelium where they create a broad-spectrum antimicrobial barrier. In certain settings, such as the urinary tract, they severely constrain microbial growth, since few visible bacteria can be found in the urine of healthy individuals. Similarly, few microbial organisms are seen in healthy bronchial secretions. In the gastrointestinal tract, home to great numbers of commensal microbes, AMPs both prevent microbes from penetrating the epithelial barrier and help shape the composition of the commensal population. Mutations can be used in humans similarly to *Drosophila* to learn about the role of AMPs. In cystic fibrosis, inactivating mutations in the cystic fibrosis transmembrane conductance regulator (CFTR) result in loss of control of the pH of the fluid layer covering the epithelium into which AMPs and their partners are secreted. As a consequence of the nonoptimal pH, the bactericidal activity of the fluid layer is reduced. Thus, loss of CFTR causes malfunction of AMPs by a change in pH. Bacteria such as Pseudomonas aeruginosa and Staphylococcus aureus become commensal, create dense biofilms, and expand to great numbers (17). Their unconstrained presence provokes the body to send in neutrophils, ultimately resulting in the destruction of bronchial tissue and lung disease. Similarly, in Crohn’s disease, the antimicrobial barrier of the ileum fails, due to malfunctioning of the Paneth cells, which normally secrete high concentrations of defensins, Reg proteins, and lysozyme onto the mucosal surface (18, 19). As a consequence, chronic inflammation ensues, resulting in progressive destruction of the ileal mucosa. Analysis of common diseases such as atopic dermatitis (20), urinary tract infection (21, 22), and periodontal disease (23) have implicated impaired expression of AMPs as contributing to the underlying pathophysiology. AMPs play a “silent” role in human health by permitting coexistence with environmental and commensal microbes.

Independently from animals, plants have also evolved cysteine-rich antimicrobial peptides (24) as an important element of innate immunity (25) with a wide spectrum of antibacterial, antifungal, insecticidal, and antiviral activities (26). There is growing evidence that plant AMPs serve an equally significant role in regulating cooperative plant-microbe interactions and colonization by beneficial microbial communities as animal AMPs. The reciprocal interplay between the plant immune system and the microbiota likely plays a critical role in shaping beneficial plant-microbiota combinations and maintaining microbial homeostasis (27).

This all indicates that the *Drosophila* study by Marra et al. (8) fits very well into the already existing observations in other organisms. The genetic approach presented provides a key step forward in concretizing the importance of AMPs in controlling microbial colonization across the tree of life.

## CONCLUDING REMARKS

**FIG 1:**
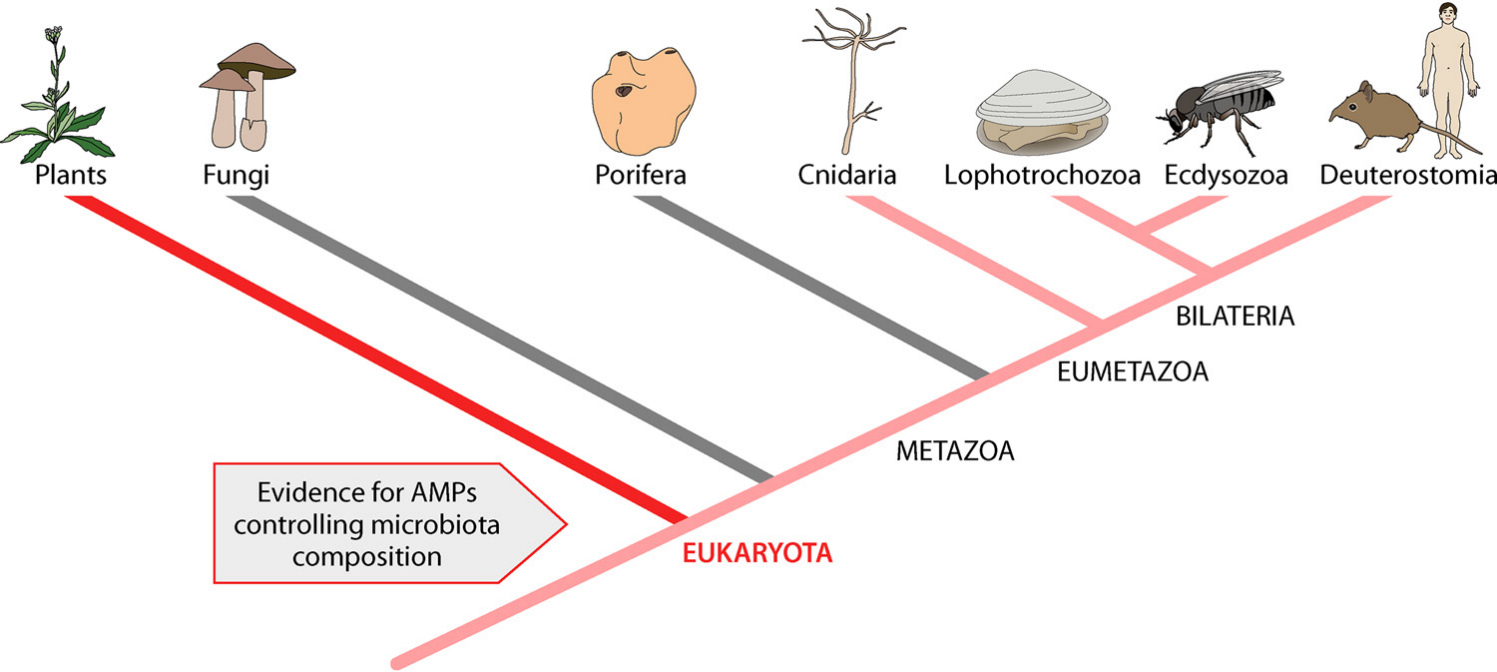
The early occurrence of AMPs and their ancestral function in controlling the microbiome. Plants and animals diverged from their protistan ancestors some 3 billion years after bacterial life originated (28). AMPs appear to serve a key function in establishing and maintaining a stable relationship with bacteria in most of the clades examined. Clades in which AMPs have been demonstrated to play a role in controlling commensal microbes are indicated by red bars. Clades in which this has not yet been discovered are indicated by gray bars.

Animal and plant evolution is intimately linked to the presence of microbes (Fig. 1). Most bacteria are not harmful to plants or animals, and many of them are beneficial, playing a key physiological or ecological role. Pathogenic and beneficial bacteria share common mechanisms by which they colonize animal tissue. From the beginning of animal and plant evolution and across the tree of life, AMPs appear to serve a crucial role in regulating the composition of the microbiome. So, AMPs do not seem to be killers so much as they are key gatekeepers to the various host tissues maintaining physiological normality of the metaorganism.
